# Trends and Species Diversity of Non-tuberculous Mycobacteria Isolated From Respiratory Samples in Northern China, 2014–2021

**DOI:** 10.3389/fpubh.2022.923968

**Published:** 2022-07-13

**Authors:** Qing Sun, Jun Yan, Xinlei Liao, Chaohong Wang, Chenqian Wang, Guanglu Jiang, Lingling Dong, Fen Wang, Hairong Huang, Guirong Wang, Junhua Pan

**Affiliations:** ^1^National Clinical Laboratory on Tuberculosis, Beijing Key Laboratory for Drug-Resistant Tuberculosis Research, Beijing Chest Hospital, Capital Medical University, Beijing Tuberculosis and Thoracic Tumor Institute, Beijing, China; ^2^Beijing Chest Hospital, Capital Medical University, Beijing Tuberculosis and Thoracic Tumor Institute, Beijing, China

**Keywords:** mycobacterium, non-tuberculous mycobacteria, species, identification, *M. intracellulare*

## Abstract

**Background:**

Pulmonary non-tuberculous mycobacteria (NTM) infection has become a public health concern in China and around the world. The objective of this study was to describe the longitudinal changes in the frequency and diversity of NTM in northern China.

**Methods:**

We retrospectively analyzed data on mycobacterium species in Beijing Chest Hospital from January 2014 to December 2021. The isolates were identified to species level by targeted DNA sequencing.

**Results:**

After excluding duplicates, 1,755 NTM strains were analyzed, which were from 27 provinces in China over 8 years. Among all mycobacteria, the proportion of NTM increased each year, from 4.24% in 2014 to 12.68% in 2021. Overall, 39 different NTM species were identified, including 23 slow growing mycobacteria (SGM) and 16 rapid growing mycobacteria (RGM). The most common species were *M. intracellulare* (51.62%), *M. abscessus* (22.22%), *M. kansasii* (8.32%), *M. avium* (7.75%) and *M. fortuitum* (2.05%). The number of NTM species identified also increased each year from 9 in 2014 to 26 in 2021. Most species showed stable isolation rates over the years; however, the proportion of *M. avium* increased from 3.85 to 10.42% during the study period. Besides, 81 non-mycobacteria strains, including *Gordonia* (21 isolates), *Nocardia* (19 isolates) and *Tsukamurella* (17 isolates), etc., were also discovered.

**Conclusion:**

The proportion of NTM and species diversity increased considerably in northern China from 2014 to 2021. *M. intracellulare* was the most common NTM isolated among respiratory specimens, followed by *M. abscessus* and *M. kansasii*. Rare NTM species and non-mycobacteria pathogens also need attention.

## Introduction

Non-tuberculous mycobacteria (NTM) can cause opportunistic infection and present a threat to public health (1). To date, more than 270 species/subspecies of NTM have been identified (http://www.bacterio.net/mycobacterium.html), and up to 60 NTM species have been proved to be human pathogens (2). The distribution of NTM species isolated from human clinical samples is geographically specific (3). As NTM differ strongly in their drug susceptibility profiles (4), understanding this diversity has significant reference value for treating and managing of these infections.

China ranked second among 30 high tuberculosis (TB) burden countries in 2020. NTM infections are prone to be misdiagnosed as multidrug-resistant TB in China, when only acid-fast staining and mycobacterial culture are used for the diagnosis of TB (5). Increased awareness of NTM identification is very important. However, precise incidence and prevalence data about NTM infection are lacking in China. Although three national TB epidemiological sampling surveys carried out in China in 1990, 2000 and 2010 reported the proportion of NTM among mycobacterial isolates, these NTM isolates were not identified to species. A few studies have reported NTM epidemiology mainly based on regional or local data (6–11). Liu et al. (12) reported the incidence of NTM in China from a national survey conducted in 2013. However, most of the reports were cross-sectional studies. The aim of this study was to determine the longitudinal changes in the frequency and diversity of NTM related to pulmonary disease over 8 years at the National Tuberculosis Clinical Laboratory of the Beijing Chest Hospital in China.

## Materials and Methods

### Data Collection

From January 2014 to December 2021, the basic patient demographic information and species identification results of positive mycobacterial cultures from respiratory samples were collected through the laboratory information system at the National Tuberculosis Clinical Laboratory of the Beijing Chest Hospital (Beijing, China).

### Smear and Culture

Direct smears were prepared and stained with auramine and examined by light-emitting diode microscopy. After processing with NALC/NaOH and centrifugation, 500 μl suspensions were inoculated into a 7 ml MGIT tube (Becton, Dickinson and Company, USA), and/or 100 μl suspensions were inoculated onto LJ medium (Encode Medical Engineering Co., Ltd, China). LJ tubes were incubated at 37 °C and examined weekly for growth for a maximum of 8 weeks and MGIT tubes were incubated in the BACTEC MGIT 960 system for 6 weeks. All of the culture-positive isolates were primarily identified as *M. tuberculosis* complex (MTBC) by MPT64 antigen testing. Isolates that were initially identified as not MTBC by the MPT64 antigen testing were further identification to the species level using target DNA sequencing.

### Species Identification

We identified the isolates to species level by target DNA sequencing, including *16S rRNA, rpoB, hsp65*, and the internal transcribed spacer region of the *16S*−*23S rRNA* region (ITS) (13, 14). Genomic DNA was isolated from isolates by boiling method. The primer sets and conditions for amplification are shown in Table 1. There were 70 mycobacterial reference strains stored in the Bio-bank in Beijing Chest Hospital (Beijing, China), which were obtained either from the American Type Culture Collection (ATCC) or from the German Collection of Microorganisms (DSM). Multigene sequence similarity for the clinical isolates was determined in comparison with the reference sequences in our Bio-bank or the multigene database using the basic local alignment search tool (BLAST). Values above 99% sequence similarity for 16S rRNA, and above 97% similarity for hsp65, rpoB and ITS genes were used for species distinction.

**Table 1:** Primers and conditions used for amplification and sequencing.

**Primers**	**Sequence (5^**′**^-3^**′**^)**	**Denaturation(s)**	**PCR conditions**		**Product length (bp)**
			**Annealing (**°**C,s)**	**Elongation (s)**	
*16S rRNA*-F	AGAGTTTGATCCTGGCTCAG	30	60, 30	40	911
*16S rRNA*-R	CCCCGTCAATTCATTTGAGTTT	30	60, 30	40	
*rpoB*-F	CGACCACTTCGGCAACCG	30	60, 30	45	351
*rpoB*-R	TCGATCGGGCACATCCGG	30	60, 30	45	
*hsp65*-F	TCGCCAAGGAGATCGAGCTGGAG	30	60, 35	40	642
*hsp65*-R	AGGTGCCGCGGATCTTGTTGAC	30	60, 35	40	
ITS -F	AAGTCGTAACAAGGTARCCG	30	60, 30	45	190–400
ITS -R	TCGCCAAGGCATCCACC	30	60, 30	45	

*ITS, the internal transcribed spacer region of the 16S-23S rRNA region*.

### Statistical Analyses

The χ2 test was used to compare differences in proportions. Statistical analysis was performed using SPSS version 22.0. Differences were considered statistically significant at *P* < 0.05.

## Results

### Changes of NTM Proportion

**Figure 1:**
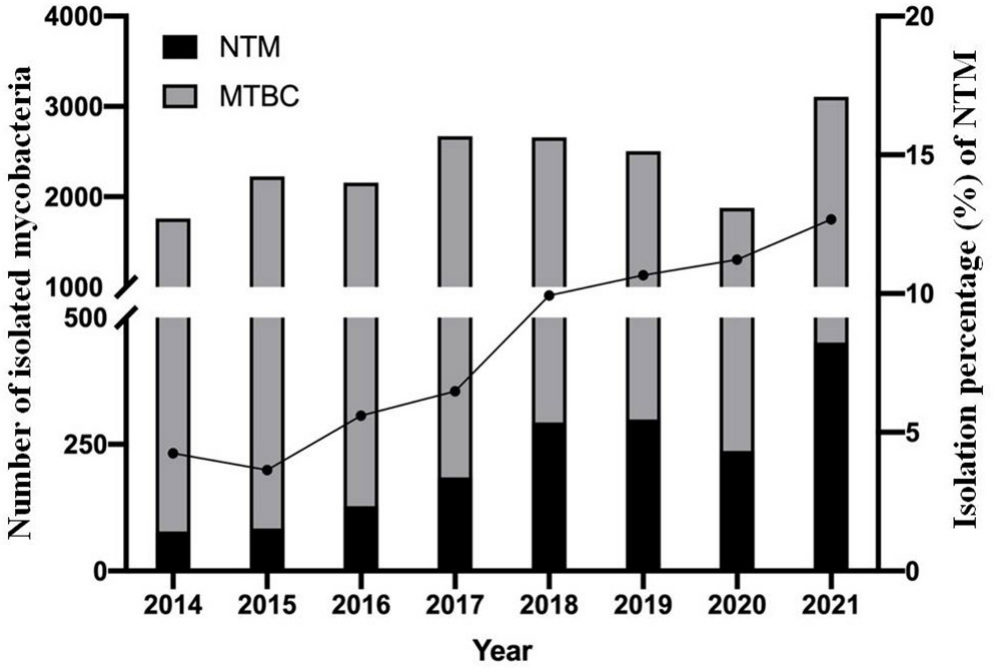
Continued upward trend in the proportion of NTM isolates over the 8-year study period. Filled circles (•) denote the percentage of NTM isolates each year. MTBC, mycobacterium tuberculosis complex; NTM, non-tuberculous mycobacteria.

A total of 20,719 mycobacterial isolates, including 1,755 NTM and 18,964 MTBC strains, were identified. The proportion of NTM among all mycobacteria increased each year, from 4.24% (78/1,839) in 2014 to 12.68% (451/3,558) in 2021 (Figure 1). The trend was statistically significant (χ^2^ = 278, *P* < 0.001); the greatest increase was from 2017 (6.48%) to 2018 (9.93%). Fewer patients were tested in 2020, due to the SARS-CoV2 pandemic started in 2019.

### The Spectrum of NTM Species

**Figure 2:**
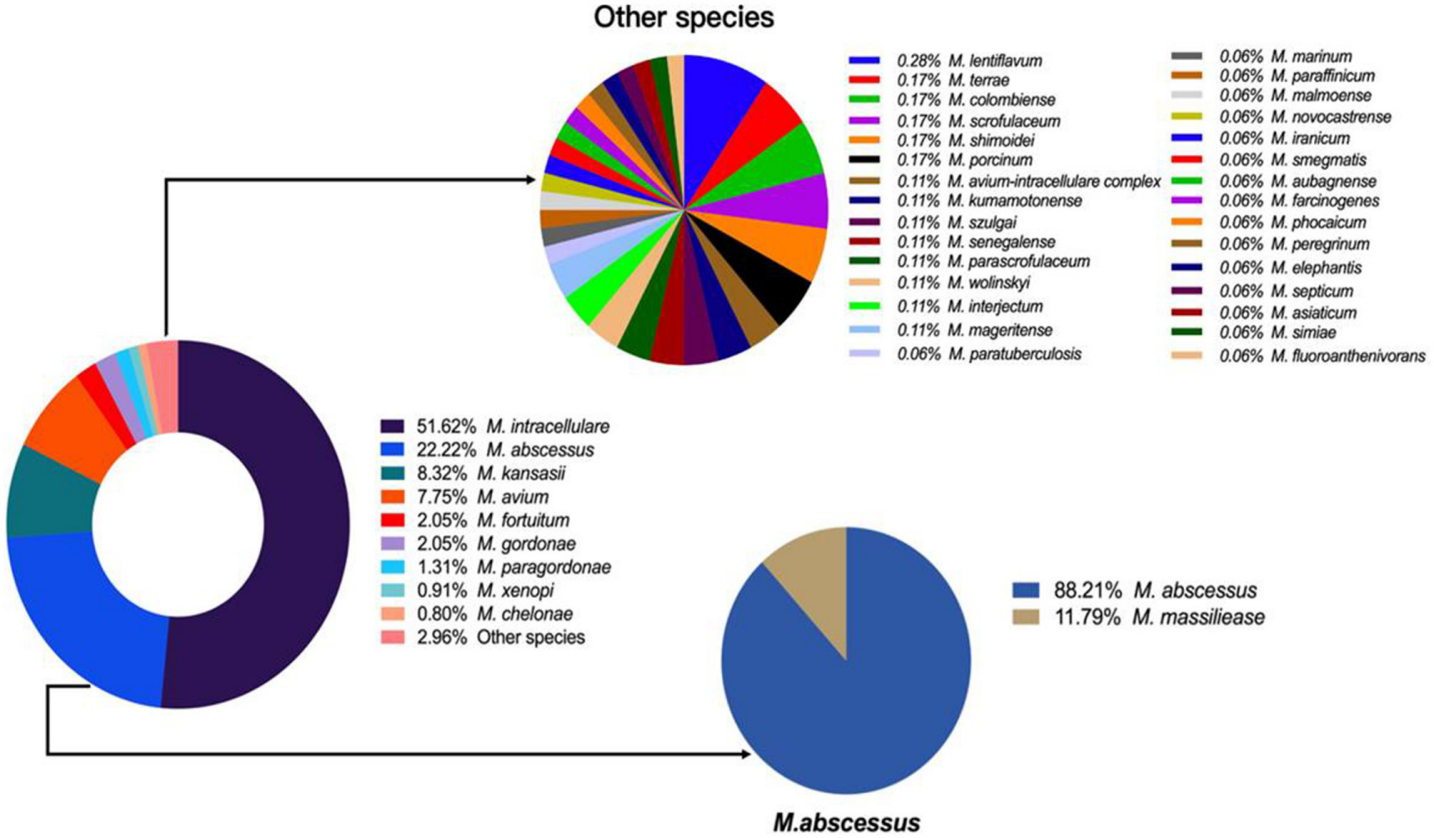
The spectrum of NTM species isolated during 2014–2021.

Overall, 39 different NTM species were identified, including 23 slow growing mycobacteria (SGM) and 16 rapid growing mycobacteria (RGM). *M. intracellulare* (51.62%), *M. abscessus* (22.22%), *M. kansasii* (8.32%), *M. avium* (7.75%) and *M. fortuitum* (2.05%) were the five most common isolated NTM, accounting for 91.96% of all NTM species (Figure 2).

**Figure 3:**
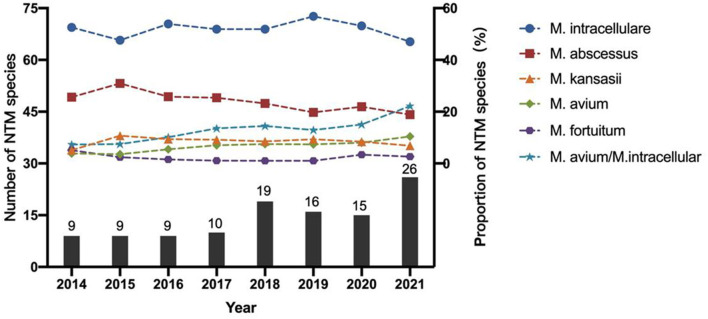
The trends of major NTM species among all the NTM strains between 2014 and 2021.

The number of NTM species identified also increased each year from 9 in 2014 to 26 in 2021 (Figure 3). *M. intracellulare* predominated in the Mycobacterium avium complex (MAC); however, the relative ratio of *M. avium* to *M. intracellulare* increased each year (Table 2; Figure 3). *M. abscessus* samples included two subspecies: *M. abscessus. abscessus* (88.21%, 344/390) and *M. abscessus. massiliense* (11.79%, 46/390). Most species showed stable isolation rates over the years; however, the proportion of *M. avium* increased from 3.85% in 2014 to 10.42% in 2021 (Figure 3).

**Table 2:** Non-tuberculous mycobacteria species isolated from respiratory specimens in China, 2014–2021.

**Species**	**2014**	**2015**	**2016**	**2017**	**2018**	**2019**	**2020**	**2021**	**Total**
*M. intracellulare*	41 (52.56)	40 (47.62)	69 (53.91)	96 (51.89)	152 (51.88)	170 (56.86)	126 (53.16)	212 (47.01)	906 (51.62)
*M. abscessus*	20 (25.64)	26 (30.95)	33 (25.78)	47 (25.41)	68 (23.21)	59 (19.73)	52 (21.94)	85 (18.85)	390 (22.22)
*M. kansasii*	4 (5.13)	9 (10.71)	12 (9.38)	17 (9.19)	25 (8.53)	28 (9.36)	20 (8.44)	31 (6.87)	146 (8.32)
*M. avium*	3 (3.85)	3 (3.57)	7 (5.47)	13 (7.03)	22 (7.51)	22 (7.36)	19 (8.02)	47 (10.42)	136 (7.75)
*M. fortuitum*	4 (5.13)	2 (2.38)	2 (1.56)	2 (1.08)	3 (1.02)	3 (1.00)	8 (3.38)	12 (2.66)	36 (2.05)
*M. gordonae*	2 (2.56)	0 (0)	0 (0)	3 (1.62)	5 (1.71)	5 (1.67)	0 (0)	21 (4.66)	36 (2.05)
*M. paragordonae*	0 (0)	0 (0)	0 (0)	1 (0.54)	2 (0.68)	4 (1.34)	1 (0.42)	15 (3.33)	23 (1.31)
*M. xenopi*	2 (2.56)	0 (0)	1 (0.78)	5 (2.70)	2 (0.68)	0 (0)	3 (1.27)	3 (0.67)	16 (0.91)
*M. chelonae*	0 (0)	1 (1.19)	1 (0.78)	0 (0)	2 (0.68)	1 (0.33)	1 (0.42)	8 (1.77)	14 (0.80)
Other species	2 (2.56)	3 (3.57)	3 (2.34)	1 (0.54)	12 (4.10)	7 (2.34)	7 (2.95)	17 (3.77)	52 (2.96)
Total	78 (4.44)	84 (4.88)	128 (7.29)	185 (10.54)	293 (16.70)	299 (17.04)	237 (13.50)	451 (25.70)	1,755 (100)

**Figure 4:**
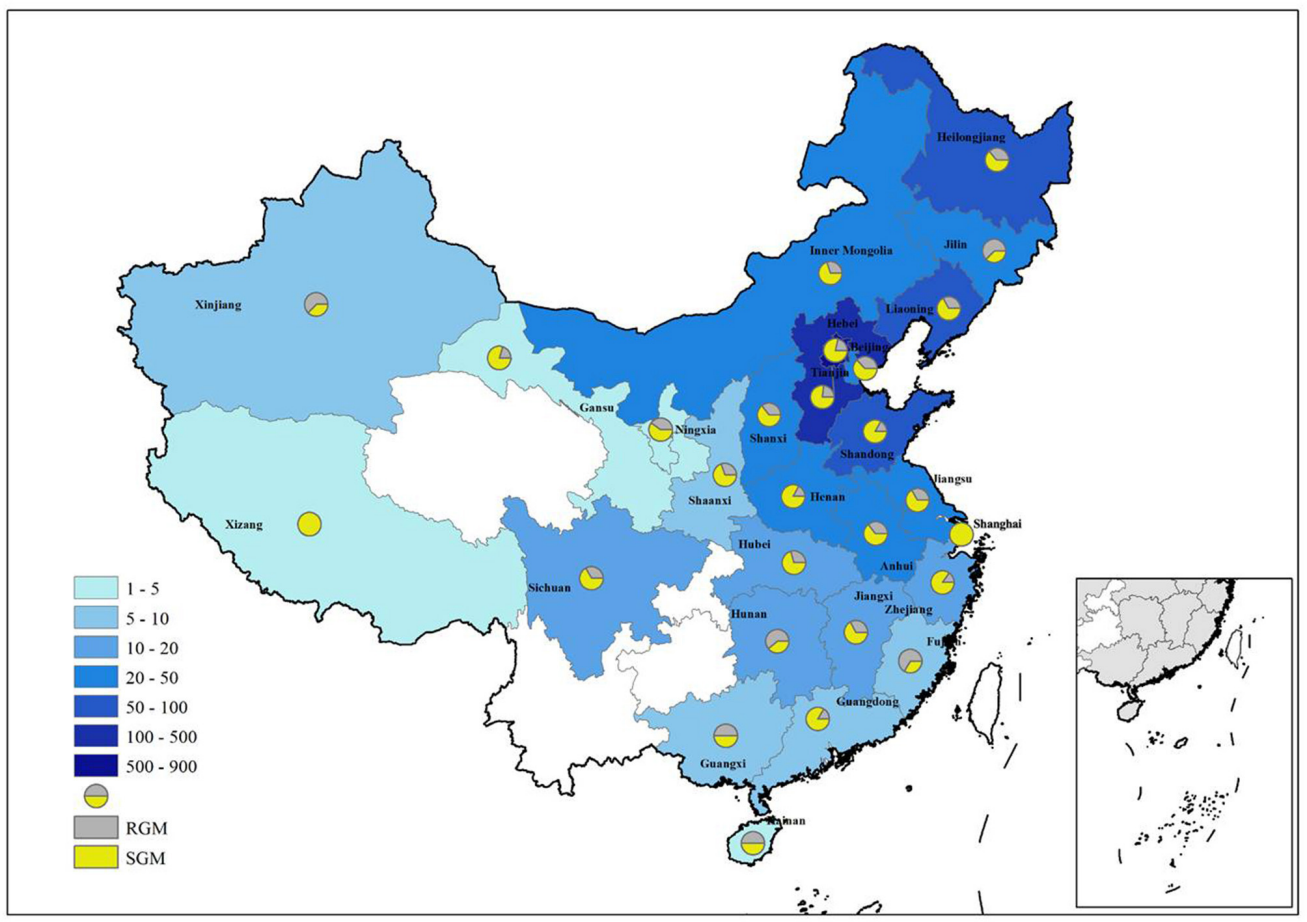
Distribution of NTM in different provinces of China. RGM rapid growing mycobacteria, SGM slow growing mycobacteria.

The 1,755 NTM isolates were from 27 provinces in China (Figure 4; Table 3). A large proportion of patients came from the north of China, with Beijing, Hebei, Liaoning, Heilongjiang, and Inner Mongolia accounting for 47.86% (840/1,755), 19.54% (343/1,755), 3.65% (64/1,755), 3.30% (58/1,755) and 2.85% (50/1,755) of all NTM identified, respectively.

**Table 3:** The distribution of clinical NTM strains throughout 27 provinces in China, 2014–2021.

**Province**	**2014**	**2015**	**2016**	**2017**	**2018**	**2019**	**2020**	**2021**	**Total**
Anhui		2	5	3	6	5	2	5	28
Beijing	40	44	54	72	122	135	110	263	840
Fujian				2	3	1	2	1	9
Gansu					2	1	1	1	5
Guangdong	1	1				3	1	2	8
Guangxi	1	2			2	2		1	8
Hainan				1		1			2
Hebei	11	7	19	38	62	67	62	77	343
Henan	2	5	5	7	7	8	3	10	47
Heilongjiang	2	2	5	6	14	8	6	15	58
Hubei	1			1	7	3	2	3	17
Hunan	3	2	2	1	4	4	1	2	19
Jilin	3	4	4	5	7	3	3	7	36
Jiangsu			4	5	2	4	3	3	21
Jiangxi			1	2	1	4	3	2	13
Liaoning	2	6	6	10	11	9	9	11	64
Inner Mongolia	2	3	5	8	10	9	4	9	50
Ningxia	1		1			2		1	5
Shandong	4	3	8	9	16	11	7	14	72
Shanxi	1	1	3	6	2	6	8	8	35
Shaanxi			1	1	2	2	1	3	10
Shanghai						1			1
Sichuan	1	1	2	4	3	2	1	1	15
Tianjin	1		2	4	5	3	5	7	27
Tibet						1			1
Xinjiang			1		2	2	1	2	8
Zhejiang	2	1			3	2	2	3	13
Total	78	8	128	185	293	299	237	451	1,755

### Non-mycobacteria Pathogens Identification

In addition to *Mycobacteria* spp., we also identified 81 non-mycobacteria strains, which were acid-fast-staining-positive or resistant to NALC-NaOH decontamination. Totally, 21 *Gordonia*, 19 *Nocardia*, 17 *Tsukamurella*, 6 *Corynebacterium*, 3 *Burkholderia*, 2 *Actinomyces* and 13 others were identified (Table 4). The similarity of the primers used to the equivalent regions in *16S rRNA, rpoB*, and *hsp65* in these species with mycobacterium was about 90, 80, and 85%, respectively.

**Table 4:** Non-mycobacteria pathogens identified from respiratory specimens in China, 2014–2021.

**Genus**	**Species**	**Total (%)**
*Gordonia* (*n* = 21)	*G. bronchialis*	9 (11.11)
	*G. sputi*	9 (11.11)
	*G. araii*	2 (2.47)
	*G. terrae*	1 (1.23)
*Nocardia* (*n* = 19)	*N. farcinica*	13 (16.05)
	*N. calcarea*	1 (1.23)
	*N. cyriacigeorgici*	1 (1.23)
	*N. wallacei*	2 (2.47)
	*N. puris*	1 (1.23)
	*N. arthritidis*	1 (1.23)
*Tsukamurella* (*n* = 17)	*T. tyrosinosolvens*	12 (14.81)
	*T. inchonensis*	2 (2.47)
	*T. pulmonis*	2 (2.47)
	*T. paurometabola*	1 (1.23)
*Corynebacterium* (*n* = 6)	*C. amycolatum*	2 (2.47)
	*C. jeikeium*	2 (2.47)
	*C. kroppenstedtii*	1 (1.23)
	*C. striatum*	1 (1.23)
*Burkholderia* (*n* = 3)		3 (3.70)
*Actinomyces* (*n* = 2)		2 (2.47)
Others (*n* = 13)		13 (16.05)
Total		81 (100)

### Demographic Data of NTM Positive Patients

**Figure 5:**
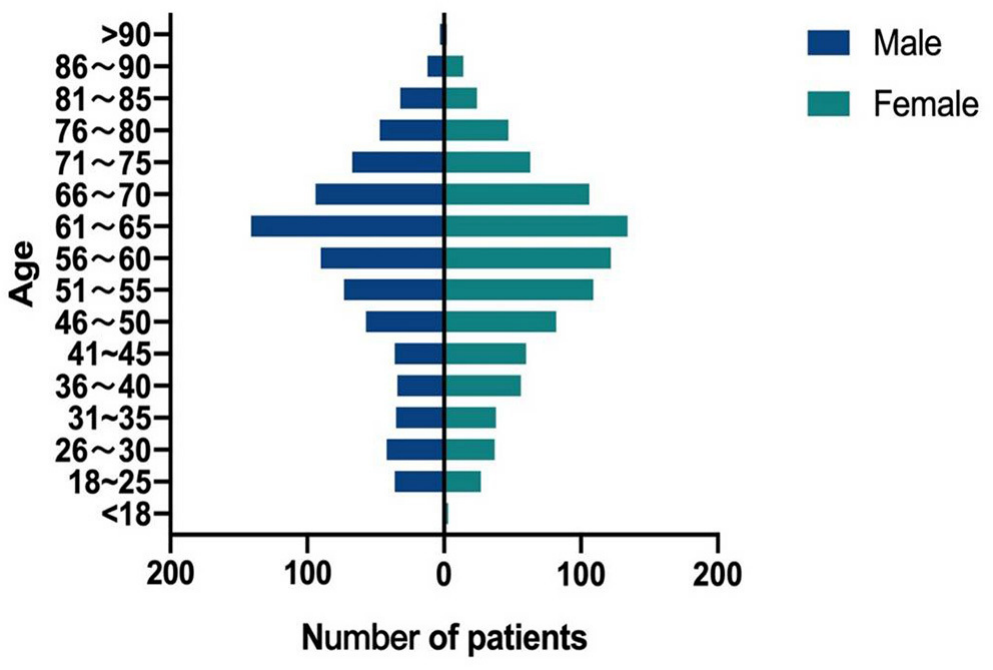
Age and gender distribution of the patients with NTM disease during 2014–20.

Thirty patients had two NTM species isolated (Table 5), among which MAC (*M. avium* or *M. intracellulare*) and MABC (*M. abscessus* or *M. massiliense*) accounting for 33.33% (10/30). The proportion of female patients in this study was 53.57% (924/1,725). The ages of the NTM patients ranged from 4 to 94 years. Patients aged 51–70 years accounted for 50.38% (869/1,725) of the total patients. In the 41–50 and 51–60 years groups, female patients had a higher proportion of NTM than male patients (*P* = 0.022) (Figure 5).

**Table 5:** NTM species found in 30 mixed infection patients in China between 2014 and 2021.

**NTM isolates**	**No. (%) of isolates**
MAC + MABC	10 (33.33)
*M. intracellulare* + *M. abscessus*	8 (26.67)
*M. intracellulare* + *M. massiliease*	1 (3.33)
*M. avium* + *M. abscessus*	1 (3.33)
MABC	6 (20.00)
*M. abscessus* + *M. massiliease*	6 (20.00)
MAC	3 (10.00)
*M. intracellulare* + *M. avium*	3 (10.00)
*M. intracellulare + M. kansasii*	2 (6.67)
*M. intracellulare + M. fortuitum*	1 (3.33)
*M. intracellulare + M.septicum*	1 (3.33)
*M. abscessus + M. kansasii*	1 (3.33)
*M. abscessus + M. fortuitum*	1 (3.33)
*M. abscessus + M. interjectum*	1 (3.33)
*M. abscessus + M. gordonae*	1 (3.33)
*M. kansasii + M. gordonae*	1 (3.33)
*M. xenopi + M. gordonae*	1 (3.33)
*M. gordonae + M. paragordonae*	1 (3.33)
Total	30 (100)

## Discussion

This study provided the NTM epidemiology in China from 2014 to 2021 when the same protocols were used for samples. Our group previously reported that the isolation rates of NTM was 2.6% (95/3,714) in respiratory samples from persons in northern China, 2008–2011 (15). The overall NTM pulmonary infection rate was 6.4% in China from a national survey conducted in 2013 (12). During our study period, the proportion of NTM almost tripled from 4.24% in 2014 to 12.68% in 2021, suggesting a considerable increase in the NTM epidemic in China.

Total 39 different NTM species were identified, indicating the diversity of NTM species circulating among patients in China. *M. intracellulare* was the most common NTM pathogen, which was similar to previous findings in Australia, Korea, India, Uruguay and so on (3, 16–19). Furthermore, we found the incidence of *M. avium* grew faster than others from 2014 to 2021, and the ratio of *M. avium* to *M. intracellulare* increased each year, suggesting a more prominent role for *M. avium* infections in China. MAC and *M. abscessus* accounted for 81.59% of the NTM strains during this study period. Beyond these common NTM species, many rare NTM species were also identified, for example *M. lentiflavum, M. terrae, M. colombiensee, M. scrofulaceum, M.shimoridei, M. porcinum, M. kumamotonense*, etc. Studies had also reported some of these species isolated from pulmonary samples (20). Precisely species identification should be conducted to facilitate pulmonary NTM treatment.

Interestingly, 81 non-mycobacteria strains including *Gordonia, Nocardia, Tsukamurella*, etc., were discovered. *Actinomycetes* with mycolic acid had been classified under genera such as *Corynebacterium, Gordonia, Mycobacterium, Nocardia, Rhodococcus, Tsukamurella, Skermania* and *Williamsia* (21, 22). The genus *Gordonia* is a gram-positive, partially acid-fast organism. *Gordonial* infections may be misdiagnosed as NTM, *Nocardia* or other actinomycetes infections due to the similar clinical manifestations (23). *Nocardia* species are gram-positive, slightly acid-fast, opportunistic pathogens. The lung is the most common infection site and symptoms of nocardiosis are similar to TB or NTM infection, such as fever, cough and chest pain (24). The genus *Tsukamurella* is an aerobic actinomycete and was a cause of opportunistic infection. *Burkholderia* are Gram-negative bacilli and opportunistic human pathogens (25–29). Several studies reported that *Gordonia, Nocardia* and *Tsukamurella* had also been identified in suspected Mycobacterium species isolates in China (8, 12, 30). In addition to NTM and MTBC infection, non-mycobacteria pathogens should also be tested when using acid-fast staining or mycobacterial culture to diagnose pulmonary disease.

Non-mycobacteria isolates could not be differentiated from NTM using traditional methods such as PNB differential media, acid-fast stains, and MPB64 protein assay. Commonly used commercial kits could only detect ~20 NTM species, and could not identify the newly discovered NTM species and non-mycobacteria pathogens (31, 32). Target DNA sequencing, such as *16S rRNA, rpoB, hsp65*, and ITS, not only could identify the NTM strains to the species level, but also could distinguish NTM from non-mycobacterial but related genera, such as *Gordonia, Nocardia* and *Tsukamurella*.

More NTM strains were isolated from female (53.57%) than from male (46.43%) in this study. The NTM patients were most common in the 51–70 (50.38%) years age group. Several reports also indicated that older women were more susceptible to NTM infection (33–35).

The large sample size, resolution power of our species identification method, and avoiding duplication were important strengths of our study, but its limitations should also be noted. First, this is a retrospective study and there may be an underestimation of the proportion of NTM. Some NTM species require specific medium requirements and low or high temperatures for growth, so some strains may be not cultivable on routine condition. Second, all of the strains were isolated from respiratory specimens. In fact respiratory samples could best reflect the distribution of NTM species in local environments. However, there may be a selection bias, as the low isolation rate of some NTM species may reflect an inability to persist in human airways. Third, the data were collected from the National Tuberculosis Clinical Laboratory of the Beijing Chest Hospital. Due to the location of Beijing Chest Hospital, most patients were from the north of China. Since the southern region had higher NTM prevalence rate than the northern region in China. Our study showed that the proportion of NTM increased considerably in northern China from 2014 to 2021, and more attention need to be taken to combat NTM.

In conclusion, the proportion of NTM and species diversity increased considerably in China from 2014 to 2021. *M. intracellulare* was the most common NTM isolated among respiratory samples, followed by *M. abscessus* and *M. kansasii*. Rare NTM species and non-mycobacteria pathogens also need attention.

## Data Availability Statement

The raw data supporting the conclusions of this article will be made available by the authors, without undue reservation.

## Author Contributions

JP, GW, and QS: substantial contributions to the conception of the work. JY, XL, ChaW, CheW, LD, and FW: acquisition and analysis of data. QS, GJ, and HH: interpretation of data. QS and GW: writing the first draft. JP and GW: revision of manuscript. QS, JY, XL, ChaW, CheW, GJ, LD, FW, HH, GW, and JP: final approval of the version to be published. All authors contributed to the article and approved the submitted version.

## Funding

This work was supported by Capital's Funds for Health Improvement and Research (2022-1G-2162), Beijing Public Health Experts Project (2022-3-040), Beijing Tongzhou Municipal Science & Technology commission (KJ2022CX044), Tongzhou Yunhe Project under Grant (YH201917), and Beijing Municipal Administration of Hospitals' Ascent Plan (DFL20181602).

## Conflict of Interest

The authors declare that the research was conducted in the absence of any commercial or financial relationships that could be construed as a potential conflict of interest.

## Publisher's Note

All claims expressed in this article are solely those of the authors and do not necessarily represent those of their affiliated organizations, or those of the publisher, the editors and the reviewers. Any product that may be evaluated in this article, or claim that may be made by its manufacturer, is not guaranteed or endorsed by the publisher.
